# Accelerometer-based prediction of ground reaction force in head-out water exercise with different exercise intensity countermovement jump

**DOI:** 10.1186/s13102-021-00389-8

**Published:** 2022-01-03

**Authors:** Kuei-Yu Chien, Wei-Gang Chang, Wan-Chin Chen, Rong-Jun Liou

**Affiliations:** 1 National Taiwan Sport University, Taoyuan City, Taiwan; 2 University of Taipei, Taipei City, Taiwan

**Keywords:** Skeletal muscle loading, Impact, External loading

## Abstract

**Background:**

Water jumping exercise is an alternative method to achieve maintenance of bone health and reduce exercise injuries. Clarifying the ground reaction force (GRF) of moderate and high cardiopulmonary exercise intensities for jumping movements can help quantify the impact force during different exercise intensities. Accelerometers have been explored for measuring skeletal mechanical loading by estimating the GRFs. Predictive regression equations for GRF using ACC on land have already been developed and performed outside laboratory settings, whereas a predictive regression equation for GRF in water exercises is not yet established. The purpose of this study was to determine the best accelerometer wear-position for three exercise intensities and develop and validate the ground reaction force (GRF) prediction equation.

**Methods:**

Twelve healthy women (23.6 ± 1.83 years, 158.2 ± 5.33 cm, 53.1 ± 7.50 kg) were recruited as participants. Triaxial accelerometers were affixed 3 cm above the medial malleolus of the tibia, fifth lumbar vertebra, and seventh cervical vertebra (C7). The countermovement jump (CMJ) cadence started at 80 beats/min and increased by 5 beats per 20 s to reach 50%, 65%, and 80% heart rate reserves, and then participants jumped five more times. One-way repeated analysis of variance was used to determine acceleration differences among wear-positions and exercise intensities. Pearson’s correlation was used to determine the correlation between the acceleration and GRF per body weight on land (GRF_V_L_BW_). Backward regression analysis was used to generate GRF_V_L_BW_ prediction equations from full models with C7 acceleration (C7 ACC), age, percentage of water deep divided by body height (PWDH), and bodyweight as predictors. Paired t-test was used to determine GRF_V_L_BW_ differences between values from the prediction equation and force plate measurement during validation. Lin’s CCC and Bland–Altman plots were used to determine the agreement between the predicted and force plate-measured GRF_V_L_BW_.

**Results:**

The raw full profile data for the resultant acceleration showed that the acceleration curve of C7 was similar to that of GRFv. The predicted formula was − 1.712 + 0.658 * C7ACC + 0.016 * PWDH + 0.008 * age + 0.003*weight. Lin’s CCC score was 0.7453, with bias of 0.369%.

**Conclusion:**

The resultant acceleration measured at C7 was identified as the valid estimated GRF_V_L_BW_ during CMJ in water.

## Background

Jumping is a general movement in many exercises and a high-impact movement mode. Studies have shown that impact exercises can effectively increase bone density [1]. However, high-impact exercises may cause burdens on the knee and hip joints or cause exercise injuries. The water’s buoyancy can reduce the impact loading to the lower-limb joints, and hydrodynamic dragging would help strengthen the lower limbs. In recent years, jumping in water has been used as a strategy to improve bone health [2] and lower limb muscle strength [3].

The take-off and landing during the jumping process are the main components that induce muscle force [4, 5]. Previous studies have found that jumping in water has a lower vertical ground reaction force (GRFv) and lower impact rate than jumping on land [4, 6]. Our previous study showed that the impact of jumping in water was lower than that on land, whereas the development intensity of jumping in the water was similar to that on land [7]. Moreover, jumping in water during the takeoff phase had a good muscle stimulation effect [4]. Therefore, water jumping exercise may be an alternative method to achieve maintenance of bone health and reduce exercise injuries.

The force plate (FP) is the gold standard for GRF measurements. However, when jumping in the water, the aqua flow affects the stability of the landing action; therefore, participants cannot accurately land inside a limited FP area consistently. Moreover, it is not possible to measure many people at the same time, which also restricts its applicability in group exercise programs. Studies indicated moderate- to high-intensity exercises to improve cardiovascular and bone benefits [8, 9]. In a previous study, the GRF at low cadence was lower than that in the sagittal plane [6]. Therefore, from a practical or clinical perspective, clarifying the GRF of moderate and high cardiopulmonary exercise intensities for jumping movements can help quantify the impact force during different exercise intensities. Accelerometers have been widely used in clinical practice to evaluate physical activity, balance control in middle-aged to older women [10–12], children [13], energy expenditure calculation, fall prediction [14], and in sport [15].

Recently, accelerometers have been explored for measuring skeletal mechanical loading by estimating the GRFs [16]. Its wear-position is mostly the tibia, ankle, and lumbar [17, 18]. Previous studies have indicated that the acceleration recorded at the upper back and lower back is a good estimate of the impact loading during continuous jumping [19, 20]. However, no current relevant studies have been conducted on jumping exercises in water. Therefore, the present study aimed to examine the acceleration and correlation between the GRF and acceleration in different accelerometer wear-positions and exercise intensities.

Several studies have also demonstrated that an accelerometer is used to calculate movement loading. Acceleration was highly correlated to the heart rate based exercise intensity [21, 22]. Predictive regression equations for GRF using ACC on land have already been developed and performed outside laboratory settings [23], whereas a predictive regression equation for GRF in water exercises is not yet established. Therefore, the secondary purpose of the present study was to develop and validate a GRF prediction equation based on the most significant wear-position in the first study.

## Materials and methods

### Participants

Twelve healthy women (23.6 ± 1.83 years, 158.2 ± 5.33 cm, 53.1 ± 7.50 kg) were recruited as participants. The study exclusion criteria were as follows: (1) participants who regularly undergo special training; (2) who underwent lower-limb reconstruction in the last 2 years; (3) serious skeletal, neurological, or muscular injury or surgery (e.g., fractures, cerebellar lesions, and stroke) history; (4) infectious skin diseases; and (5) fear of water after adaptation. Participants were asked not to exercise vigorously or perform lower-limb training 1 week before each experimental session to prevent fatigue from affecting the experimental results. The present study was reviewed and approved by the Fu Jun Catholic University Human Research Ethics Committee of Taiwan (C105016). The present study conforms to the Declaration of Helsinki for studies involving humans.

### Testing procedure

Each participant performed a regular stretching exercise, as well as stretching the quadriceps, biceps femoris, gastrocnemius, tibialis anterior, gluteus medius, and iliopsoas. Before starting the experiment, three triaxial accelerometers (TSD109F, Biopac Systems Inc., USA) were affixed to the right leg ankle, lumbar, and neck using double-sided tape. The position was 3 cm above the medial malleolus of the tibia (TA), fifth lumbar vertebra (L5), and seventh cervical vertebra (C7). The best-represented sensor placement was located as close as possible to the center of the body mass, such as the sternum for the whole-body movement analysis [24]. The device was placed at the center of the upper back to minimize artifact movements between the device and the body [25]. A waterproof film was covered on accelerometers. Finally, the accelerometer was fixed with a light elastic band. Participants wore watersport shoes when they performed countermovement jump (CMJ) testing in the water. Heart rate was measured using the Garmin Forerunner 920XT (Garmin HRM-Tri, Garmin Ltd, Taiwan) with a surface electrode chest strap. The sample rate was 1 Hz. Wireless frequency/ protocol: 2.4 GHz @ + 1 dBm nominal. Participants stood on land for 5 min. The average of the last 1 min was the resting heart rate on land, and then the participants stood in the water for 5 min and the average of the last 1 min was the resting heart rate in the water. The predicted maximum heart rate (MHR _predicted_) targeted was calculated by the formula 206.9 − (0.67*age) [26]. The MHR in water was calculated as the predicted MHR minus the difference between RHR measured on land and RHR measured in water [27]: MHR_water_ = MHR _predicted_ − △HR, where △HR = RHR_land_ − RHR_water_. We used Karvonen Formula to calculate the 50, 65, 80% HR reserve (HRR) as target HR_water_ = [MHR_water_ − RHR_water_] × 50, 65, 80% + RHR_water_. Afterward, participants were asked to complete the 8–10 continuous CMJ at 80 beats per minute on a 464 × 508-mm waterproof force plate (OR6-WP, AMTI; Watertown, USA) to ensure that their movements were correct. The environmental temperature of the water-based test was 31–33 °C, and the water depth was 1 m. We used a metronome to set the cadence of the movement. Participants must follow the instructions of the exercise instructor to perform the correct CMJ movements at the set cadence. In the formal testing, the participant’s jumping cadence started at 80 beats/min and increased by 5 beats per 20 s to approach 50% heart rate reserve (HRR). When the participant’s HRR reached 50%, they jumped five more times and then walked slowly to rest until they reach 50% HRR again. After resting, jump cadence started with the number of beats reaching 50% HRR and then increased by 5 beats per 20 s to reach 65% HRR. When they reached 65% HRR, they jumped five more times and then walked slowly to rest until they reach 50% HRR again. The jump testing cadence started with the number of beats approaching 65% HRR and then increased by 5 beats per 20 s to reach 80% HRR. When the HRR approached 80%, the test was completed after another five jumps. The jumping number calculated from each testing phase started to approach the target heart rate after jumping more than five times.

### Data collection and analysis

The acceleration signals were obtained using three accelerometers (fixed on C7, L5, and TA). Force data were integrated using an acquisition system (Biopac Systems Inc., USA) and AcqKnowledge 4.2 software. The sampling frequency of signals was 1000 Hz. All signals were filtered through Matlab 2017a software using a sixth-order low-pass filter with a cut-off frequency of 50 Hz. The resultant acceleration was calculated using three axes of each accelerometer. Subsequently, the vertical ground reaction force data of the force plate were used to segment the landing period of each jump (the vertical GRF was > 10% of the subject’s water body weight). Finally, to exclude the influence of buoyancy on gravity in water, the GRFv in water was standardized using body weight on land as GRF_V_L_BW_.

### Statistical analysis

Data are expressed as the mean ± standard deviation. One-way repeated analysis of variance was used to determine the difference in acceleration among the three wear-positions at the same exercise intensity and among the three exercise intensities at the same wear-position. Pearson’s correlation was used to analyze the correlation between acceleration and GRF_V_L_BW_. We confirmed the best wear-position according to the above statistical results, and two-thirds of data were randomly allocated into the equation development data DD (N = 1041), and one-third of the data was the validation data (VD) (N = 538). An independent sample t-test was used to determine the difference in outcome variables between DD and VD. Backward regression analysis was used to generate GRF_V_L_BW_ prediction equations from the full models with C7 ACC, age, percentage of water deep divided by body height (PWDH), and body weight as possible predictors. A paired t-test was used to determine the differences in GRF_V_L_BW_ between values derived from prediction equations and force plate measurement in VD. Bland–Altman plots were used to determine the agreement between the C7ACC and predicted and force plate measured GRF_V_L_BW_. All analyses were performed using the SPSS version 20.0 (IBM, Chicago, IL), and statistical significance was set at α = 0.05.

## Results

**Fig. 1:**
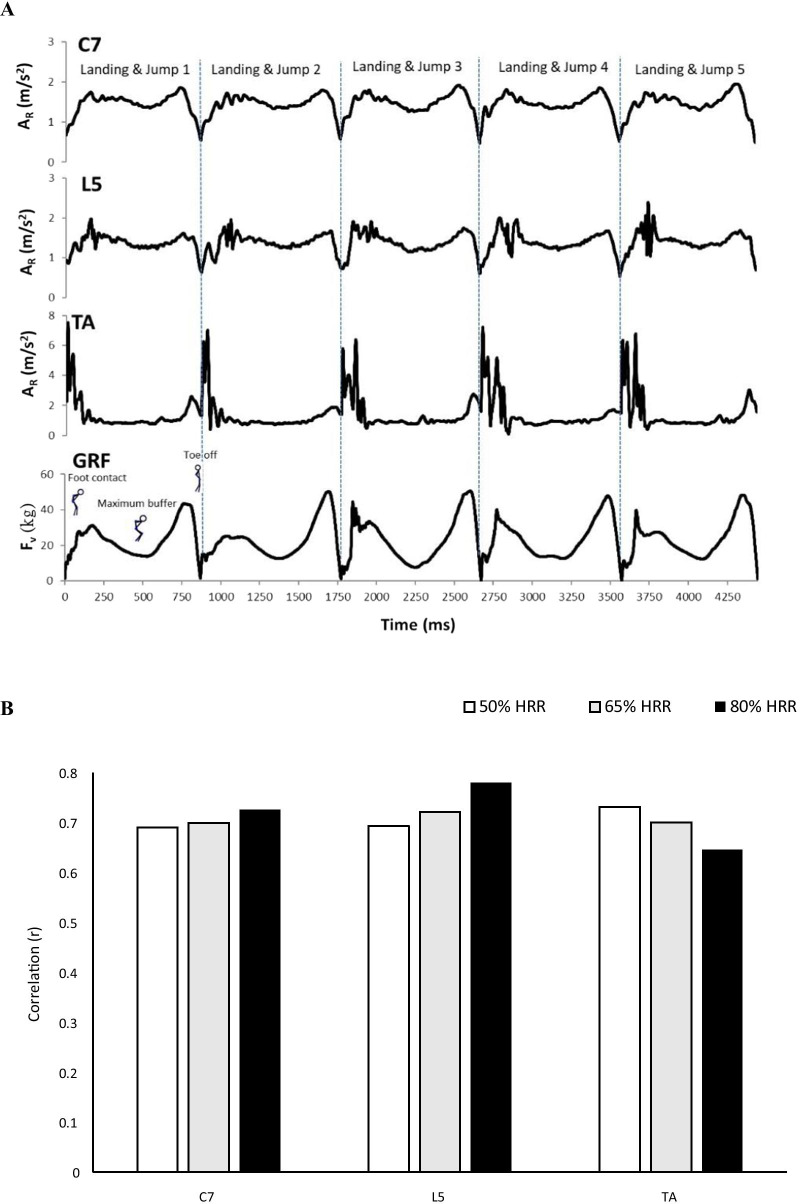
Acceleration and vertical ground reaction force of continuous countermovement jumps in an aquatic environment. **A** Raw full profile data for resultant acceleration and vertical ground reaction force without flight phase. **B** The correlation of GRF_V_L_BW_ and acceleration in different wear positions with various exercise intensities

The raw full profile data for the resultant acceleration showed that the acceleration curve of C7 was similar to that of GRFv. Different interface interferences were observed to induce noise signals during the jumping process at the L5 wear-position. The TA accelerometer had a large signal during landing (Fig. 1A). Under the same exercise intensity, the acceleration of L5 was significantly lower than that of C7 and TA (Table 1). No differences were observed between C7 and TA. When accelerometers were worn at C7 and L5, the acceleration at 80% HRR was higher than that at 50% HRR. However, the acceleration was similar for the three intensities at the TA position. The GRF_V_L_BW_ of 80% and 65% HRR was significantly higher than the 50% HRR. No difference was observed between 80 and 65% and between 50 and 65% HRR. The correlation between GRF_V_L_BW_ per jump and acceleration slightly increased with an increase in exercise intensity at the C7 and C5 wear-positions (Fig. 1B). Conversely, the correlation of GRF_V_L_BW_ per jump and acceleration decreased as exercise intensity increased at the TA wear-position.

**Table 1:** Comparison of different acceleration position, GRF_V_L_BW_ per jump and jumping numbers in different exercise intensity

	C7	L5	TA	GRF_V_L_BW_	JN
50% HRR	1.51 ± 0.099	1.47 ± 0.0920^#^	1.55 ± 0.158^§^	0.64 ± 0.088	34.0 ± 2.88
65% HRR	1.53 ± 0.105	1.48 ± 0.095^#^	1.56 ± 0.140	0.67 ± 0.075	45.3 ± 3.74*
80% HRR	1.61 ± 0.125^*^	1.55 ± 0.119^*#^	1.65 ± 0.162^§^	0.73 ± 0.099^*^	52.7 ± 3.23*

**C7:** the seventh cervical vertebra**; L5:** the fifth lumbar vertebra; TA: 3 cm above the medial malleolus of tibia; GRF_V_L_BW:_ ground reaction force per body weight on land. JN: jumping numbers

^*^Significantly different from 50% HRR (*p* < .05)

^#^Significantly different from C7 under same exercise intensity (*p* < .05)

^§^Significantly different from L5 under same exercise intensity (*p* < .05)

The present study showed no differences in age (23.7±1.95 vs.23.6±1.85 yrs, *p* = 0.308), BMI (21.2 ± 2.04 vs. 21.2 ± 2.10, *p* = 0.904), GRF_V_L_BW_ (0.6837 ± 0.1112 vs. 0.6828 ± 0.01121 BW, *p* = 0.888) and C7ACC (1.562 ± 0.1249 vs.1.568 ± 0.1240 g, *p* = 0.304). According to the stepwise regression statistical method, we found that C7ACC the percentage of deep water divided by body height, age, and body weight were important parameters for predicting GRF_V_L_BW_. The prediction formula was − 1.712 + 0.658* C7ACC + 0.016*PWDH + 0.008*age + 0.003*weight. That showed a moderate-high adjusted R^2^ of 0.592. Data from VG were applied to cross-validate the prediction equations. We found no difference between the C7 ACC-predicted GRF_V_L_BW_ values and the force plate measured values (Table 2).

**Table 2:** The GRF_V_L_BW_ between the values derived from the prediction equations and measurement in validation data

C7 ACC-predicted values		Force plate -measured values		*p* value
Mean	SD	Mean	SD	
0.679	0.0861	0.683	0.1121	0.206

**Fig. 2:**
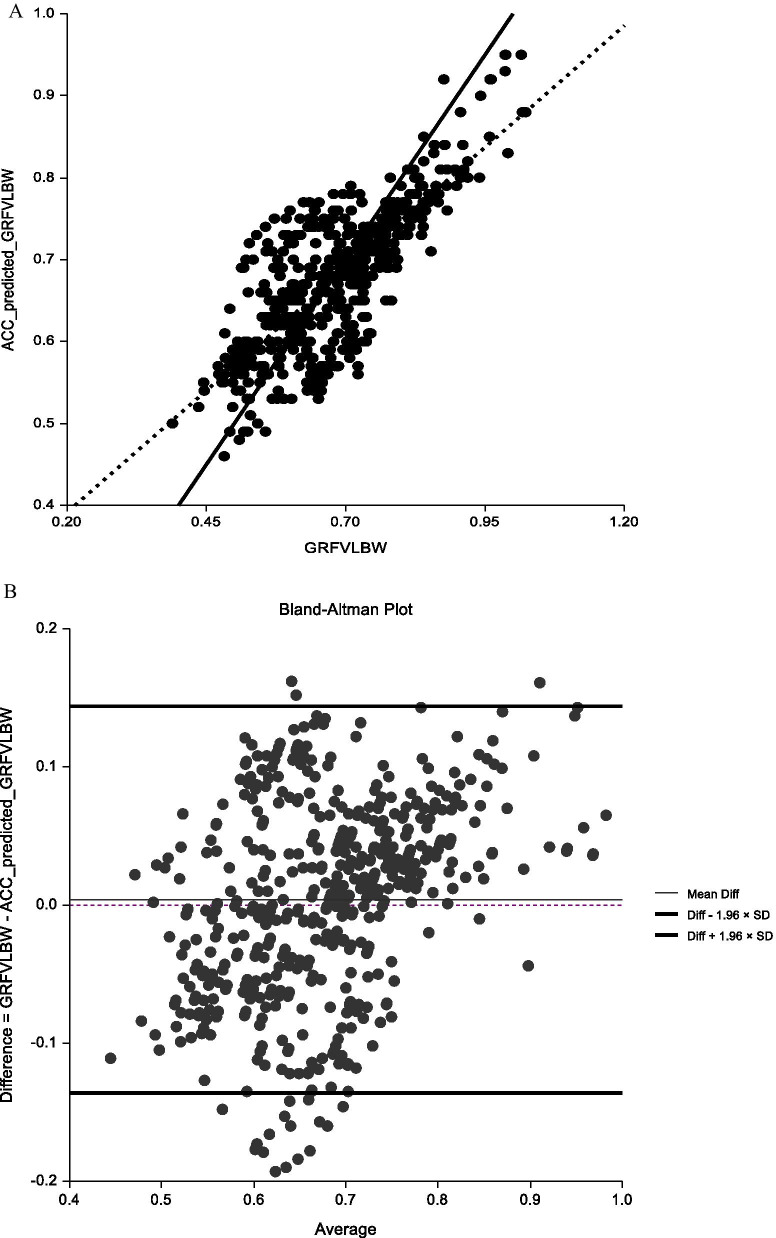
Intraclass correlation coefficient and the C7 ACC prediction equation from validation data. **A** Lin's Concordance Correlation Coefficient graphs for GRF_V_L_BW_ comparison. The solid line represents the 45-degree line of perfect agreement through the origin, while the dotted line is the line of best fit. The spread of data and angle of the line of best fit in the 50–80% HRR (n = 538) illustrate that agreement between ACC predicted is stronger compared to the GRF_V_L_BW_. **B** Bland–Altman plot of GRF_V_L_BW_ measured by force plate and predicted by the C7 ACC prediction equation

A previous study indicated CCCs of 0.51–0.70 were considered moderately positively correlated and 0.71–0.90 as a good positive correlation [28]. Figure 2A shows that Lin’s CCC of 0.7453 showed a high correlation. The Bland–Altman plots display the individual participant differences in the validation group between the measured and predicted GRF_V_L_BW_ against the mean measured and predicted GRF_V_L_BW_ (Fig. 2B). Each Bland–Altman plot displays the mean difference (dashed line) and 95% confidence interval (± 2SD; dotted lines). A strong agreement was found between the measured and predicted values of GRF_V_L_BW_. The bias percentage was 0.353.

## Discussion

The full profile data without the flight phase showed that the acceleration pattern worn at the C7 position was similar to that of the force plate, including the peaks of landing and take-off. A previous study showed that wear-position at the hip estimates walking and running GRFs without a force plate [23]. Linear mixed models suggested that 24–50% of the variability in peak GRF and loading rates could be attributed to measured accelerations at the hip [29]. The graphics at the L5 position were also similar to those of the force plate. However, the acceleration signal was affected when preparing to take off after landing. In tibial acceleration, a previous study showed small to moderate associations between ankle-based outcomes and the corresponding GRF and LR while walking and jogging [29]. Elvin’s study also indicated that a tibial accelerometer can be used to determine the ground reaction forces experienced during jump landing [22]. However, the raw full profile data showed that the acceleration was lower than the GRF during the push-off stage. This was consistent with the results of our study. The landing phase showed an obvious acceleration during CMJ jumping. Nearly no changes were observed in the acceleration of the other phases of CMJ jumping. Such a curve was very different from the GRF curve, and its representativeness was insufficient.

The acceleration of L5 was significantly lower than that of C7 and TA for the three exercise intensities. The main reason for this is that the knee joint bends to buffer the landing impact during the landing phase. Furthermore, the L5 was close to the center of mass of the body. Whether it is the truck flexion angle during landing or the extension angle during takeoff, it is not as large as compared to that in C7 at the distal end of the body. A previous study demonstrated that the acceleration at the Sacrum (i.e., near L5) during jumping was lower than the segmental kinematic centroid [15], which was consistent with the lower acceleration at L5 position in the present study. A previous study indicated the sensor was attached too close to a center of rotation; thus, the amplitude of the resulting measured signal might be attenuated [30]. When sagittal flexion and extension are performed at the distal part of the body, “whiplash motion” should be greater than the proximal end. Moreover, Simons et al. indicated that women have more subcutaneous fat around the lower back area than men [20]. A higher amount of subcutaneous fat affects the signal. This is a potential explanation for the acceleration recorded on the upper back more accurately during drop landing.

Alberton’s study indicated that the GRF of frontal hops increased with increasing cadence [31]. The CMJ is similar to the frontal hop. Therefore, we observed that the correlation between GRF_V_L_BW_ and acceleration was high (r = 0.69–0.78) when participants jumped in three exercise intensities in the L5 and C7 wear-positions. The number of jumps was also found to be increased with increased exercise intensity (Table 1). The acceleration correlation of the TA did not increase with increasing exercise intensity but decreased with increasing exercise intensity because the jumping height and landing impact decreased as the frequency increased. Therefore, the correlation was low with increasing exercise intensity. According to the above description, C7 was selected as the target wear-position to process the GRF_V_L_BW_ prediction regression based on the following reasons: (1) the raw full profile data for the resultant acceleration showed that the curve of C7 ACC was similar to that of GRFv; (2) when the accelerometer was worn at C7, the acceleration at 80% HRR was higher than that at 50% HRR, a finding consistent with the results of GRF_V_L_BW_; and (3) the acceleration was highly positively correlated with GRF_V_L_BW_ per jump. The correlation did not decrease with increasing exercise intensity.

The present study showed that C7 ACC, PWDH, body weight, and age were the major factors in predicting GRF_V_L_BW._ A previous study showed that as the water level increased, the percentage of water level to height increased and its buoyancy increased [32]. The greater the buoyancy product, the smaller the landing impact. This will also affect the GRFv during take-off. Further, bodyweight is an important factor that affects impact. The heavier the weight, the greater the impact. The regression formula presented in the present study was different from the regression formula of previous studies, showing that only bodyweight and device acceleration are the main influencing factors and predictive variables. PWDH is an important predictive parameter of GRF_V_L_BW_ in water. PWDH parameters in the GRF_V_L_BW_ prediction variable helped quantify the GRF_V_L_BW_ for participants of different body heights or different water depths.

CCCs of 0.51–0.70 were considered moderately positively correlated and 0.71–0.90 good positive correlation [28]. Figure 2A showed that Lin’s CCC was moderately to highly correlate. The present study showed that the values did not reach 95% (Fig. 2B), where GRF_V_L_BW_ data measured by the force plate were low and the ACC estimated value was high. The resulting difference was negative and fell outside − 1.96*SD. This may be due to the increased exercise intensity and faster jumping speed. Controlling the falling motion within the 464 × 508-mm force plate is difficult, resulting in data deviation. Although 19 data points fall outside − 1.96*SD (3.53%), the percentage bias was only 0.396%, which still has application potential.

## Limitation

The first limitation was that the present study used five more jumps when the target heart rate was attained because of the short time required to approach the target hearing rate. Therefore, the steady-state response of the target exercise intensity could not be achieved. We considered the practical application scenarios of high-intensity intermittent jumping and the workload tolerance of participants; therefore, a continuous jump test of different intensities with intermediate low-intensity rest was adopted. In a previous study, water jumping research was a gradually increasing test that maintained a speed [31]. However, the testing mode was more in line with the design feature of the short duration of high-intensity intermittent jumping exercises in the water [33]. Aging is one of the factors affecting exercise performance. The second limitation of the present study was that participants were young women. There may be limitations if the present study results are directly applied to middle-aged women. However, the GRF_V_L_BW_ estimation formula parameters, age, and C7ACC parameters may solve the influence of aging. In current study, machine learning (ML) was used to analyzing swimming accelerometer data to recognize swimming style, count stokes and so on [34]. We suggested that can use ML techniques to improve the accuracy of the prediction model in the future study. Moreover, for the results of the present study to be applicable to men, further studies on men are needed. Finally, we recommend that water jumping research be conducted on middle-aged women with a high prevalence of cardiovascular and osteoporosis diseases in the future study. These results should be more representative and applicable.

## Conclusions

The resultant acceleration measured at C7 was identified as the valid estimated GRF_V_L_BW_ for jumping skeletal loading in the water. The predicted formula was − 1.712 + 0.658 * C7ACC + 0.016 * PWDH + 0.008 * age + 0.003 * weight.

## Data Availability

The datasets used and/or analyzed during the current study are available from the corresponding author on reasonable request.

## Abbreviations

C7: The seventh cervical vertebra
C7 ACC: C7 acceleration
FP: Force plate
GRF: Ground reaction force
GRFv: Vertical ground reaction force
CMJ: Countermovement jump
GRF_V_L_BW_: GRF per body weight on land
HRR: Heart rate reserve
L5: Fifth lumbar vertebra
JN: Jumping numbers
PWDH: Percentage of water deep divided by body height
TA: The medial malleolus of the tibia
